# A tutorial on sensitivity analyses in clinical trials: the what, why, when and how

**DOI:** 10.1186/1471-2288-13-92

**Published:** 2013-07-16

**Authors:** Lehana Thabane, Lawrence Mbuagbaw, Shiyuan Zhang, Zainab Samaan, Maura Marcucci, Chenglin Ye, Marroon Thabane, Lora Giangregorio, Brittany Dennis, Daisy Kosa, Victoria Borg Debono, Rejane Dillenburg, Vincent Fruci, Monica Bawor, Juneyoung Lee, George Wells, Charles H Goldsmith

**Affiliations:** 1 Department of Clinical Epidemiology and Biostatistics, McMaster University, Hamilton, ON, Canada; 2 Departments of Pediatrics and Anesthesia, McMaster University, Hamilton, ON, Canada; 3 Center for Evaluation of Medicine, St Joseph’s Healthcare Hamilton, Hamilton, ON, Canada; 4 Biostatistics Unit, Father Sean O’Sullivan Research Center, St Joseph’s Healthcare Hamilton, Hamilton, ON, Canada; 5 Population Health Research Institute, Hamilton Health Sciences, Hamilton, ON, Canada; 6 Department of Psychiatry and Behavioral Neurosciences, McMaster University, Hamilton, ON, Canada; 7 Population Genomics Program, McMaster University, Hamilton, ON, Canada; 8 GSK, Mississauga, ON, Canada; 9 Department of Kinesiology, University of Waterloo, Waterloo, ON, Canada; 10 Department of Nephrology, Toronto General Hospital, Toronto, ON, Canada; 11 Department of Pediatrics, McMaster University, Hamilton, ON, Canada; 12 Michael G. DeGroote School of Medicine, McMaster University, Hamilton, ON, Canada; 13 McMaster Integrative Neuroscience Discovery & Study (MiNDS) Program, McMaster University, Hamilton, ON, Canada; 14 Department of Biostatistics, Korea University, Seoul, Korea; 15 Department of Clinical Epidemiology, University of Ottawa, Ottawa, ON, Canada; 16 Faculty of Health Sciences, Simon Fraser University, Burnaby, BC, Canada

**Keywords:** Sensitivity analysis, Clinical trials, Robustness

## Abstract

**Background:**

Sensitivity analyses play a crucial role in assessing the robustness of the findings or conclusions based on primary analyses of data in clinical trials. They are a critical way to assess the impact, effect or influence of key assumptions or variations—such as different methods of analysis, definitions of outcomes, protocol deviations, missing data, and outliers—on the overall conclusions of a study.

The current paper is the second in a series of tutorial-type manuscripts intended to discuss and clarify aspects related to key methodological issues in the design and analysis of clinical trials.

**Discussion:**

In this paper we will provide a detailed exploration of the key aspects of sensitivity analyses including: 1) what sensitivity analyses are, why they are needed, and how often they are used in practice; 2) the different types of sensitivity analyses that one can do, with examples from the literature; 3) some frequently asked questions about sensitivity analyses; and 4) some suggestions on how to report the results of sensitivity analyses in clinical trials.

**Summary:**

When reporting on a clinical trial, we recommend including planned or *posthoc* sensitivity analyses, the corresponding rationale and results along with the discussion of the consequences of these analyses on the overall findings of the study.

## Background

The credibility or interpretation of the results of clinical trials relies on the validity of the methods of analysis or models used and their corresponding assumptions. An astute researcher or reader may be less confident in the findings of a study if they believe that the analysis or assumptions made were not appropriate. For a primary analysis of data from a prospective randomized controlled trial (RCT), the key questions for investigators (and for readers) include:

• How confident can I be about the results?

• Will the results change if I change the definition of the outcome (e.g., using different cut-off points)?

• Will the results change if I change the method of analysis?

• Will the results change if we take missing data into account? Will the method of handling missing data lead to different conclusions?

• How much influence will minor protocol deviations have on the conclusions?

• How will ignoring the serial correlation of measurements within a patient impact the results?

• What if the data were assumed to have a non-Normal distribution or there were outliers?

• Will the results change if one looks at subgroups of patients?

• Will the results change if the full intervention is received (i.e. degree of compliance)?

The above questions can be addressed by performing sensitivity analyses—testing the effect of these “changes” on the observed results. If, after performing sensitivity analyses the findings are consistent with those from the primary analysis and would lead to similar conclusions about treatment effect, the researcher is reassured that the underlying factor(s) had little or no influence or impact on the primary conclusions. In this situation, the results or the conclusions are said to be “robust”.

The objectives of this paper are to provide an overview of how to approach sensitivity analyses in clinical trials. This is the second in a series of tutorial-type manuscripts intended to discuss and clarify aspects related to some key methodological issues in the design and analysis of clinical trials. The first was on pilot studies [1]. We start by describing what sensitivity analysis is, why it is needed and how often it is done in practice. We then describe the different types of sensitivity analyses that one can do, with examples from the literature. We also address some of the commonly asked questions about sensitivity analysis and provide some guidance on how to report sensitivity analyses.

## Discussion

### Sensitivity Analysis

#### What is a sensitivity analysis in clinical research?

Sensitivity Analysis (SA) is defined as “a method to determine the robustness of an assessment by examining the extent to which results are affected by changes in methods, models, values of unmeasured variables, or assumptions” with the aim of identifying “results that are most dependent on questionable or unsupported assumptions” [2]. It has also been defined as “a series of analyses of a data set to assess whether altering any of the assumptions made leads to different final interpretations or conclusions” [3]. Essentially, SA addresses the “what-if-the-key-inputs-or-assumptions-changed”-type of question. If we want to know whether the results change when something about the way we approach the data analysis changes, we can make the change in our analysis approach and document the changes in the results or conclusions. For more detailed coverage of SA, we refer the reader to these references [4-7].

#### Why is sensitivity analysis necessary?

The design and analysis of clinical trials often rely on assumptions that may have some effect, influence or impact on the conclusions if they are not met. It is important to assess these effects through sensitivity analyses. Consistency between the results of primary analysis and the results of sensitivity analysis may strengthen the conclusions or credibility of the findings. However, it is important to note that the definition of consistency may depend in part on the area of investigation, the outcome of interest or even the implications of the findings or results.

It is equally important to assess the robustness to ensure appropriate interpretation of the results taking into account the things that may have an impact on them. Thus, it imperative for every analytic plan to have some sensitivity analyses built into it.

The United States (US) Food and Drug Administration (FDA) and the European Medicines Association (EMEA), which offer guidance on Statistical Principles for Clinical Trials, state that “it is important to evaluate the robustness of the results and primary conclusions of the trial.” Robustness refers to “the sensitivity of the overall conclusions to various limitations of the data, assumptions, and analytic approaches to data analysis” [8]. The United Kingdom (UK) National Institute of Health and Clinical Excellence (NICE) also recommends the use of sensitivity analysis in “exploring alternative scenarios and the uncertainty in cost-effectiveness results” [9].

#### How often is sensitivity analysis reported in practice?

To evaluate how often sensitivity analyses are used in medical and health research, we surveyed the January 2012 editions of major medical journals (British Medical Journal, New England Journal of Medicine, the Lancet, Journal of the American Medical Association and the Canadian Medical Association Journal) and major health economics journals (Pharmaco-economics, Medical Decision making, European Journal of Health Economics, Health Economics and the Journal of Health Economics). From every article that included some form of statistical analyses, we evaluated: i) the percentage of published articles that reported results of some sensitivity analyses; and ii) the types of sensitivity analyses that were performed. Table 1 provides a summary of the findings. Overall, the point prevalent use of sensitivity analyses is about 26.7% (36/135) —which seems very low. A higher percentage of papers published in health economics than in medical journals (30.8% vs. 20.3%) reported some sensitivity analyses. Among the papers in medical journals, 18 (28.1%) were RCTs, of which only 3 (16.6%) reported sensitivity analyses. Assessing robustness of the findings to different methods of analysis was the most common type of sensitivity analysis reported in both types of journals. Therefore despite their importance, sensitivity analyses are under-used in practice. Further, sensitivity analyses are more common in health economics research—for example in conducting cost-effectiveness analyses, cost-utility analyses or budget-impact analyses—than in other areas of health or medical research.

**Table 1:** Comparison of sensitivity analyses reported in medical and health economics journals in January 2012

**Variable**	**Medical journals**	**Health economics journals**
Number with statistical analysis	64^$^	71
Number with sensitivity analysis (%)	13^&^ (20.3)	22 (30.9)
Type of sensitivity analysis		
• Methods of analysis	5	12
• Outcome definitions	4	1
• Distributional assumptions	1	0
• Key assumptions*	2	4
• Missing data	1	4
• Baseline imbalances	0	1

^$^Eighteen (18) of these were randomized controlled trials, of which only 3 reported sensitivity analyses.

^&^Of which 3 were randomized controlled trials and 10 were observational studies.

*Assumptions related to the participants, interventions or outcomes that can affect the results of the trial. For example, considering a second episode of cancer as a relapse instead of a continuation of the first; in a cost-effectiveness analysis, modifying the anticipated frequency of the intervention.

### Types of sensitivity analyses

In this section, we describe scenarios that may require sensitivity analyses, and how one could use sensitivity analyses to assess the robustness of the statistical analyses or findings of RCTs. These are not meant to be exhaustive, but rather to illustrate common situations where sensitivity analyses might be useful to consider (Table 2). In each case, we provide examples of actual studies where sensitivity analyses were performed, and the implications of these sensitivity analyses.

**Table 2:** Examples of common scenarios for sensitivity analyses in clinical trials

**Scenario**	**Sensitivity analysis options**
Outliers	- Assess outlier by z-score or boxplot
	- Perform analyses with and without outliers
Non-compliance or protocol violation in RCTs	Perform
	- Intention-to-treat analysis (as primary analysis)
	- As-treated analysis
	- Per-protocol analysis
Missing data	- Analyze only complete cases
	- Impute the missing data using single or multiple imputation methods and redo the analysis
Definitions of outcomes	- Perform analyses on outcomes of different cut-offs or definitions
Clustering or correlation	- Compare the analysis that ignores clustering with one primary method chosen to account for clustering
and multi-center trials	
	- Compare the analysis that ignores clustering with several methods of accounting for clustering [10,11]
	- Perform analysis with and without adjusting for center
	- Use different methods of adjusting for center [12]
Competing risks in RCTs	- Perform a survival analysis for each event separately
	- Use a proportional sub-distribution hazard model (Fine & Grey approach)
	- Fit one model by taking into account all the competing risks together [13]
Baseline imbalance	Perform:
	- Analysis with and without adjustment for baseline characteristics
	- Analysis with different methods of adjusting for baseline imbalance. e.g. Multivariable regression vs. propensity score method
Distributional assumptions	Perform analyses under different distributional assumptions
	- Different distributions (e.g. Poisson vs. Negative binomial)
	- Parametric vs. non-parametric methods
	- Classical vs. Bayesian methods
	- Different prior distributions

#### Impact of outliers

An outlier is an observation that is numerically distant from the rest of the data. It deviates markedly from the rest of the sample from which it comes [14,15]. Outliers are usually exceptional cases in a sample. The problem with outliers is that they can deflate or inflate the mean of a sample and therefore influence any estimates of treatment effect or association that are derived from the mean. To assess the potential impact of outliers, one would first assess whether or not any observations meet the definition of an outlier—using either a boxplot or z-scores [16]. Second, one could perform a sensitivity analysis with and without the outliers.

Examples:

• In a cost–utility analysis of a practice-based osteopathy clinic for subacute spinal pain, Williams et al. reported lower costs per quality of life year ratios when they excluded outliers [17]. In other words, there were certain participants in the trial whose costs were very high, and were making the average costs look higher than they probably were in reality. The observed cost per quality of life year was not robust to the exclusion of outliers, and changed when they were excluded.

• A primary analysis based on the intention-to-treat principle showed no statistically significant differences in reducing depression between a nurse-led cognitive self-help intervention program compared to standard care among 218 patients hospitalized with angina over 6 months. Some sensitivity analyses in this trial were performed by excluding participants with high baseline levels of depression (outliers) and showed a statistically significant reduction in depression in the intervention group compared to the control. This implies that the results of the primary analysis were affected by the presence of patients with baseline high depression [18].

#### Impact of non-compliance or protocol deviations

In clinical trials some participants may not adhere to the intervention they were allocated to receive or comply with the scheduled treatment visits. Non-adherence or non-compliance is a form of protocol deviation. Other types of protocol deviations include switching between intervention and control arms (i.e. treatment switching or crossovers) [19,20], or not implementing the intervention as prescribed (i.e. intervention fidelity) [21,22].

Protocol deviations are very common in interventional research [23-25]. The potential impact of protocol deviations is the dilution of the treatment effect [26,27]. Therefore, it is crucial to determine the robustness of the results to the inclusion of data from participants who deviate from the protocol. Typically, for RCTs the primary analysis is based on an intention-to-treat (ITT) principle—in which participants are analyzed according to the arm to which they were randomized, irrespective of whether they actually received the treatment or completed the prescribed regimen [28,29]. Two common types of sensitivity analyses can be performed to assess the robustness of the results to protocol deviations: 1) per-protocol (PP) analysis—in which participants who violate the protocol are excluded from the analysis [30]; and 2) as-treated (AT) analysis—in which participants are analyzed according to the treatment they actually received [30]. The PP analysis provides the ideal scenario in which all the participants comply, and is more likely to show an effect; whereas the ITT analysis provides a “real life” scenario, in which some participants do not comply. It is more conservative, and less likely to show that the intervention is effective. For trials with repeated measures, some protocol violations which lead to missing data can be dealt with alternatively. This is covered in more detail in the next section.

Examples:

• A trial was designed to investigate the effects of an electronic screening and brief intervention to change risky drinking behaviour in university students. The results of the ITT analysis (on all 2336 participants who answered the follow-up survey) showed that the intervention had no significant effect. However, a sensitivity analysis based on the PP analysis (including only those with risky drinking at baseline and who answered the follow-up survey; n = 408) suggested a small beneficial effect on weekly alcohol consumption [31]. A reader might be less confident in the findings of the trial because of the inconsistency between the ITT and PP analyses—the ITT was not robust to sensitivity analyses. A researcher might choose to explore differences in the characteristics of the participants who were included in the ITT versus the PP analyses.

• A study compared the long-term effects of surgical versus non-surgical management of chronic back pain. Both the ITT and AT analyses showed no significant difference between the two management strategies [32]. A reader would be more confident in the findings because the ITT and AT analyses were consistent—the ITT was robust to sensitivity analyses.

#### Impact of missing data

Missing data are common in every research study. This is a problem that can be broadly defined as “missing some information on the phenomena in which we are interested” [33]. Data can be missing for different reasons including (1) non-response in surveys due to lack of interest, lack of time, nonsensical responses, and coding errors in data entry/transfer; (2) incompleteness of data in large data registries due to missing appointments, not everyone is captured in the database, and incomplete data; and (3) missingness in prospective studies as a result of loss to follow up, dropouts, non-adherence, missing doses, and data entry errors.

The choice of how to deal with missing data would depend on the mechanisms of missingness. In this regard, data can be missing at random (MAR), missing not at random (MNAR), or missing completely at random (MCAR). When data are MAR, the missing data are dependent on some other observed variables rather than any unobserved one. For example, consider a trial to investigate the effect of pre-pregnancy calcium supplementation on hypertensive disorders in pregnancy. Missing data on the hypertensive disorders is dependent (conditional) on being pregnant in the first place. When data are MCAR, the cases with missing data may be considered a random sample drawn from all the cases. In other words, there is no “cause” of missingness. Consider the example of a trial comparing a new cancer treatment to standard treatment in which participants are followed at 4, 8, 12 and 16 months. If a participant misses the follow up at the 8th and 16th months and these are unrelated to the outcome of interest, in this case mortality, then this missing data is MCAR. Reasons such as a clinic staff being ill or equipment failure are often unrelated to the outcome of interest. However, the MCAR assumption is often challenging to prove because the reason data is missing may not be known and therefore it is difficult to determine if it is related to the outcome of interest. When data are MNAR, missingness is dependent on some unobserved data. For example, in the case above, if the participant missed the 8th month appointment because he was feeling worse or the 16th month appointment because he was dead, the missingness is dependent on the data not observed because the participant was absent. When data are MAR or MCAR, they are often referred to as ignorable (provided the cause of MAR is taken into account). MNAR on the other hand, is nonignorable missingness. Ignoring the missingness in such data leads to biased parameter estimates [34]. Ignoring missing data in analyses can have implications on the reliability, validity and generalizability of research findings.

The best way to deal with missing data is prevention, by steps taken in the design and data collection stages, some of which have been described by Little et al. [35]. But this is difficult to achieve in most cases. There are two main approaches to handling missing data: i) ignore them—and use complete case analysis; and ii) impute them—using either single or multiple imputation techniques. Imputation is one of the most commonly used approaches to handling missing data. Examples of single imputation methods include hot deck, cold deck method, mean imputation, regression technique, last observation carried forward (LOCF) and composite methods—which uses a combination of the above methods to impute missing values. Single imputation methods often lead to biased estimates and under-estimation of the true variability in the data. Multiple imputation (MI) technique is currently the best available method of dealing with missing data under the assumption that data are missing at random (MAR) [33,36-38]. MI addresses the limitations of single imputation by using multiple imputed datasets which yield unbiased estimates, and also accounts for the within- and between-dataset variability. Bayesian methods using statistical models that assume a prior distribution for the missing data can also be used to impute data [35].

It is important to note that ignoring missing data in the analysis would be implicitly assuming that the data are MCAR, an assumption that is often hard to verify in reality.

There are some statistical approaches to dealing with missing data that do not necessarily require formal imputation methods. For example, in studies using continuous outcomes, linear mixed models for repeated measures are used for analyzing outcomes measured repeatedly over time [39,40]. For categorical responses or count data, generalized estimating equations [GEE] and random-effects generalized linear mixed models [GLMM] methods may be used [41,42]. In these models it is assumed that missing data are MAR. If this assumption is valid, then the complete-case analysis by including predictors of missing observations will provide consistent estimates of the parameter.

The choice of whether to ignore or impute missing data, and how to impute it, may affect the findings of the trial. Although one approach (ignore or impute, and if the latter, how to impute) should be made *a priori,* a sensitivity analysis can be done with a different approach to see how “robust” the primary analysis is to the chosen method for handling missing data.

Examples:

• A 2011 paper reported the sensitivity analyses of different strategies for imputing missing data in cluster RCTs with a binary outcome using the community hypertension assessment trial (CHAT) as an example. They found that variance in the treatment effect was underestimated when the amount of missing data was large and the imputation strategy did not take into account the intra-cluster correlation. However, the effects of the intervention under various methods of imputation were similar. The CHAT intervention was not superior to usual care [43].

• In a trial comparing methotrexate with to placebo in the treatment of psoriatic arthritis, the authors reported both an intention-to-treat analysis (using multiple imputation techniques to account for missing data) and a complete case analysis (ignoring the missing data). The complete case analysis, which is less conservative, showed some borderline improvement in the primary outcome (psoriatic arthritis response criteria), while the intention-to-treat analysis did not [44]. A reader would be less confident about the effects of methotrexate on psoriatic arthritis, due to the discrepancy between the results with imputed data (ITT) and the complete case analysis.

#### Impact of different definitions of outcomes (e.g. different cut-off points for binary outcomes)

Often, an outcome is defined by achieving or not achieving a certain level or threshold of a measure. For example in a study measuring adherence rates to medication, levels of adherence can be dichotomized as achieving or not achieving at least 80%, 85% or 90% of pills taken. The choice of the level a participant has to achieve can affect the outcome—it might be harder to achieve 90% adherence than 80%. Therefore, a sensitivity analysis could be performed to see how redefining the threshold changes the observed effect of a given intervention.

Examples:

• In a trial comparing caspofungin to amphotericin B for febrile neutropoenic patients, a sensitivity analysis was conducted to investigate the impact of different definitions of fever resolution as part of a composite endpoint which included: resolution of any baseline invasive fungal infection, no breakthrough invasive fungal infection, survival, no premature discontinuation of study drug, and fever resolution for 48 hours during the period of neutropenia. They found that response rates were higher when less stringent fever resolution definitions were used, especially in low-risk patients. The modified definitions of fever resolution were: no fever for 24 hours before the resolution of neutropenia; no fever at the 7-day post-therapy follow-up visit; and removal of fever resolution completely from the composite endpoint. This implies that the efficacy of both medications depends somewhat on the definition of the outcomes [45].

• In a phase II trial comparing minocycline and creatinine to placebo for Parkinson’s disease, a sensitivity analysis was conducted based on another definition (threshold) for futility. In the primary analysis a predetermined futility threshold was set at 30% reduction in mean change in Unified Parkinson’s Disease Rating Scale (UPDRS) score, derived from historical control data. If minocycline or creatinine did not bring about at least a 30% reduction in UPDRS score, they would be considered as futile and no further testing will be conducted. Based on the data derived from the current control (placebo) group, a new threshold of 32.4% (more stringent) was used for the sensitivity analysis. The findings from the primary analysis and the sensitivity analysis both confirmed that that neither creatine nor minocycline could be rejected as futile and should both be tested in Phase III trials [46]. A reader would be more confident of these robust findings.

#### Impact of different methods of analysis to account for clustering or correlation

Interventions can be administered to individuals, but they can also be administered to clusters of individuals, or naturally occurring groups. For example, one might give an intervention to students in one class, and compare their outcomes to students in another class – the class is the cluster. Clusters can also be patients treated by the same physician, physicians in the same practice center or hospital, or participants living in the same community. Likewise, in the same trial, participants may be recruited from multiple sites or centers. Each of these centers will represent a cluster. Patients or elements within a cluster often have some appreciable degree of homogeneity as compared to patients between clusters. In other words, members of the same cluster are more likely to be similar to each other than they are to members of another cluster, and this similarity may then be reflected in the similarity or correlation measure, on the outcome of interest.

There are several methods of accounting or adjusting for similarities within clusters, or “clustering” in studies where this phenomenon is expected or exists as part of the design (e.g., in cluster randomization trials). Therefore, in assessing the impact of clustering one can build into the analytic plans two forms of sensitivity analyses: i) analysis with and without taking clustering into account—comparing the analysis that ignores clustering (i.e. assumes that the data are independent) to one primary method chosen to account for clustering; ii) analysis that compares several methods of accounting for clustering.

Correlated data may also occur in longitudinal studies through repeat or multiple measurements from the same patient, taken over time or based on multiple responses in a single survey. Ignoring the potential correlation between several measurements from an individual can lead to inaccurate conclusions [47].

Here are a few references to studies that compared the outcomes that resulted when different methods were/were not used to account for clustering. Noteworthy, is the fact that the analytical approaches for cluster-RCTs and multi-site RCTs are similar.

Examples:

• Ma et al. performed sensitivity analyses of different methods of analysing cluster RCTs [48]. In this paper they compared three cluster-level methods (un-weighted linear regression, weighted linear regression and random-effects meta-regression) to six individual level analysis methods (standard logistic regression, robust standard errors approach, GEE, random effects meta-analytic approach, random-effects logistic regression and Bayesian random-effects regression). Using data from the CHAT trial, in this analysis, all nine methods provided similar results, re-enforcing the hypothesis that the CHAT intervention was not superior to usual care.

• Peters et al. conducted sensitivity analyses to compare different methods—three cluster-level (un-weighted regression of practice log odds, regression of log odds weighted by their inverse variance and random-effects meta-regression of log odds with cluster as a random effect) and five individual-level methods (standard logistic regression ignoring clustering, robust standard errors, GEE, random-effects logistic regression and Bayesian random-effects logistic regression.)—for analyzing cluster randomized trials using an example involving a factorial design [13]. In this analysis, they demonstrated that the methods used in the analysis of cluster randomized trials could give varying results, with standard logistic regression ignoring clustering being the least conservative.

• Cheng et al. used sensitivity analyses to compare different methods (six models for clustered binary outcomes and three models for clustered nominal outcomes) of analysing correlated data in discrete choice surveys [49]. The results were robust to various statistical models, but showed more variability in the presence of a larger cluster effect (higher within-patient correlation).

• A trial evaluated the effects of lansoprazole on gastro-esophageal reflux disease in children from 19 clinics with asthma. The primary analysis was based on GEE to determine the effect of lansoprazole in reducing asthma symptoms. Subsequently they performed a sensitivity analysis by including the study site as a covariate. Their finding that lansoprazole did not significantly improve symptoms was robust to this sensitivity analysis [50].

• In addition to comparing the performance of different methods to estimate treatment effects on a continuous outcome in simulated multicenter randomized controlled trials [12], the authors used data from the Computerization of Medical Practices for the Enhancement of Therapeutic Effectiveness (COMPETE) II [51] to assess the robustness of the primary results (based on GEE to adjust for clustering by provider of care) under different methods of adjusting for clustering. The results, which showed that a shared electronic decision support system improved care and outcomes in diabetic patients, were robust under different methods of analysis.

#### Impact of competing risks in analysis of trials with composite outcomes

A competing risk event happens in situations where multiple events are likely to occur in a way that the occurrence of one event may prevent other events from being observed [48]. For example, in a trial using a composite of death, myocardial infarction or stroke, if someone dies, they cannot experience a subsequent event, or stroke or myocardial infarction—death can be a competing risk event. Similarly, death can be a competing risk in trials of patients with malignant diseases where thrombotic events are important. There are several options for dealing with competing risks in survival analyses: (1) to perform a survival analysis for each event separately, where the other competing event(s) is/are treated as censored; the common representation of survival curves using the Kaplan-Meier estimator is in this context replaced by the cumulative incidence function (CIF) which offers a better interpretation of the incidence curve for one risk, regardless of whether the competing risks are independent; (2) to use a proportional sub-distribution hazard model (Fine & Grey approach) in which subjects that experience other competing events are kept in the risk set for the event of interest (i.e. as if they could later experience the event); (3) to fit one model, rather than separate models, taking into account all the competing risks together (Lunn-McNeill approach) [13]. Therefore, the best approach to assessing the influence of a competing risk would be to plan for sensitivity analysis that adjusts for the competing risk event.

Examples:

• A previously-reported trial compared low molecular weight heparin (LMWH) with oral anticoagulant therapy for the prevention of recurrent venous thromboembolism (VTE) in patients with advanced cancer, and a subsequent study presented sensitivity analyses comparing the results from standard survival analysis (Kaplan-Meier method) with those from competing risk methods—namely, the cumulative incidence function (CIF) and Gray's test [52]. The results using both methods were similar. This strengthened their confidence in the conclusion that LMWH reduced the risk of recurrent VTE.

• For patients at increased risk of end stage renal disease (ESRD) but also of premature death not related to ESRD, such as patients with diabetes or with vascular disease, analyses considering the two events as different outcomes may be misleading if the possibility of dying before the development of ESRD is not taken into account [49]. Different studies performing sensitivity analyses demonstrated that the results on predictors of ESRD and death for any cause were dependent on whether the competing risks were taken into account or not [53,54], and on which competing risk method was used [55]. These studies further highlight the need for a sensitivity analysis of competing risks when they are present in trials.

#### Impact of baseline imbalance in RCTs

In RCTs, randomization is used to balance the expected distribution of the baseline or prognostic characteristics of the patients in all treatment arms. Therefore the primary analysis is typically based on ITT approach unadjusted for baseline characteristics. However, some residual imbalance can still occur by chance. One can perform a sensitivity analysis by using a multivariable analysis to adjust for hypothesized residual baseline imbalances to assess their impact on effect estimates.

Examples:

• A paper presented a simulation study where the risk of the outcome, effect of the treatment, power and prevalence of the prognostic factors, and sample size were all varied to evaluate their effects on the treatment estimates. Logistic regression models were compared with and without adjustment for the prognostic factors. The study concluded that the probability of prognostic imbalance in small trials could be substantial. Also, covariate adjustment improved estimation accuracy and statistical power [56].

• In a trial testing the effectiveness of enhanced communication therapy for aphasia and dysarthria after stroke, the authors conducted a sensitivity analysis to adjust for baseline imbalances. Both primary and sensitivity analysis showed that enhanced communication therapy had no additional benefit [57].

#### Impact of distributional assumptions

Most statistical analyses rely on distributional assumptions for observed data (e.g. Normal distribution for continuous outcomes, Poisson distribution for count data, or binomial distribution for binary outcome data). It is important not only to test for goodness-of-fit for these distributions, but to also plan for sensitivity analyses using other suitable distributions. For example, for continuous data, one can redo the analysis assuming a Student-T distribution—which is symmetric, bell-shaped distribution like the Normal distribution, but with thicker tails; for count data, once can use the Negative-binomial distribution—which would be useful to assess the robustness of the results if over-dispersion is accounted for [52]. Bayesian analyses routinely include sensitivity analyses to assess the robustness of findings under different models for the data and prior distributions [58]. Analyses based on parametric methods—which often rely on strong distributional assumptions—may also need to be evaluated for robustness using non-parametric methods. The latter often make less stringent distributional assumptions. However, it is essential to note that in general non-parametric methods are less efficient (i.e. have less statistical power) than their parametric counter-parts if the data are Normally distributed.

Examples:

• Ma et al. performed sensitivity analyses based on Bayesian and classical methods for analysing cluster RCTs with a binary outcome in the CHAT trial. The similarities in the results after using the different methods confirmed the results of the primary analysis: the CHAT intervention was not superior to usual care [10].

• A negative binomial regression model was used [52] to analyze discrete outcome data from a clinical trial designed to evaluate the effectiveness of a pre-habilitation program in preventing functional decline among physically frail, community-living older persons. The negative binomial model provided an improved fit to the data than the Poisson regression model. The negative binomial model provides an alternative approach for analyzing discrete data where over-dispersion is a problem [59].

### Commonly asked questions about sensitivity analyses

• Q: Do I need to adjust the overall level of significance for performing sensitivity analyses?

A: No. Sensitivity analysis is typically a re-analysis of either the same outcome using different approaches, or different definitions of the outcome—with the primary goal of assessing how these changes impact the conclusions. Essentially everything else including the criterion for statistical significance needs to be kept constant so that we can assess whether any impact is attributable to underlying sensitivity analyses.

• Q: Do I have to report all the results of the sensitivity analyses?

A: Yes, especially if the results are different or lead to different a conclusion from the original results—whose sensitivity was being assessed. However, if the results remain robust (i.e. unchanged), then a brief statement to this effect may suffice.

• Q: Can I perform sensitivity analyses posthoc?

A: It is desirable to document all planned analyses including sensitivity analyses in the protocol *a priori*. Sometimes, one cannot anticipate all the challenges that can occur during the conduct of a study that may require additional sensitivity analyses. In that case, one needs to incorporate the anticipated sensitivity analyses in the statistical analysis plan (SAP), which needs to be completed before analyzing the data. Clear rationale is needed for every sensitivity analysis. This may also occur *posthoc*.

• Q: How do I choose between the results of different sensitivity analyses? (i.e. which results are the best?)

A: The goal of sensitivity analyses is not to select the “best” results. Rather, the aim is to assess the robustness or consistency of the results under different methods, subgroups, definitions, assumptions and so on. The assessment of robustness is often based on the magnitude, direction or statistical significance of the estimates. You cannot use the sensitivity analysis to choose an alternate conclusion to your study. Rather, you can state the conclusion based on your primary analysis, and present your sensitivity analysis as an example of how confident you are that it represents the truth. If the sensitivity analysis suggests that the primary analysis is not robust, it may point to the need for future research that might address the source of the inconsistency. Your study cannot answer the question *which results are best?* To answer the question of *which method is best and under what conditions,* simulation studies comparing the different approaches on the basis of bias, precision, coverage or efficiency may be necessary.

• Q: When should one perform sensitivity analysis?

A: The default position should be to plan for sensitivity analysis in every clinical trial. Thus, all studies need to include some sensitivity analysis to check the robustness of the primary findings. All statistical methods used to analyze data from clinical trials rely on assumptions—which need to either be tested whenever possible, with the results assessed for robustness through some sensitivity analyses. Similarly, missing data or protocol deviations are common occurrences in many trials and their impact on inferences needs to be assessed.

• Q: How many sensitivity analyses can one perform for a single primary analysis?

A: The number is not an important factor in determining what sensitivity analyses to perform. The most important factor is the rationale for doing any sensitivity analysis. Understanding the nature of the data, and having some content expertise are useful in determining which and how many sensitivity analyses to perform. For example, varying the ways of dealing with missing data is unlikely to change the results if 1% of data are missing. Likewise, understanding the distribution of certain variables can help to determine which cut points would be relevant. Typically, it is advisable to limit sensitivity analyses to the primary outcome. Conducting multiple sensitivity analysis on all outcomes is often neither practical, nor necessary.

• Q: How many factors can I vary in performing sensitivity analyses?

A: Ideally, one can study the impact of all key elements using a factorial design—which would allow the assessment of the impact of individual and joint factors. Alternatively, one can vary one factor at a time to be able to assess whether the factor is responsible for the resulting impact (if any). For example, in a sensitivity analysis to assess the impact of the Normality assumption (analysis assuming Normality e.g. T-test vs. analysis without assuming Normality e.g. Based on a sign test) and outlier (analysis with and without outlier), this can be achieved through 2x2 factorial design.

• Q: What is the difference between secondary analyses and sensitivity analyses?

A: Secondary analyses are typically analyses of secondary outcomes. Like primary analyses which deal with primary outcome(s), such analyses need to be documented in the protocol or SAP. In most studies such analyses are exploratory—because most studies are not powered for secondary outcomes. They serve to provide support that the effects reported in the primary outcome are consistent with underlying biology. They are different from sensitivity analyses as described above.

• Q: What is the difference between subgroup analyses and sensitivity analyses?

A: Subgroup analyses are intended to assess whether the effect is similar across specified groups of patients or modified by certain patient characteristics [60]. If the primary results are statistically significant, subgroup analyses are intended to assess whether the observed effect is consistent across the underlying patient subgroups—which may be viewed as some form of sensitivity analysis. In general, for subgroup analyses one is interested in the results for each subgroup, whereas in subgroup “sensitivity” analyses, one is interested in the similarity of results across subgroups (ie. robustness across subgroups). Typically subgroup analyses require specification of the subgroup hypothesis and rationale, and performed through inclusion of an interaction term (i.e. of the subgroup variable x main exposure variable) in the regression model. They may also require adjustment for alpha—the overall level of significance. Furthermore, most studies are not usually powered for subgroup analyses.

## Conclusion

### Reporting of sensitivity analyses

There has been considerable attention paid to enhancing the transparency of reporting of clinical trials. This has led to several reporting guidelines, starting with the CONSORT Statement [61] in 1996 and its extensions [http://www.equator-network.org]. Not one of these guidelines specifically addresses how sensitivity analyses need to be reported. On the other hand, there is some guidance on how sensitivity analyses need to be reported in economic analyses [62]—which may partly explain the differential rates of reporting of sensitivity analyses shown in Table 1. We strongly encourage some modifications of all reporting guidelines to include items on sensitivity analyses—as a way to enhance their use and reporting. The proposed reporting changes can be as follows:

• In Methods Section: Report the planned or *posthoc* sensitivity analyses and rationale for each.

• In Results Section: Report whether or not the results of the sensitivity analyses or conclusions are similar to those based on primary analysis. If similar, just state that the results or conclusions remain robust. If different, report the results of the sensitivity analyses along with the primary results.

• In Discussion Section: Discuss the key limitations and implications of the results of the sensitivity analyses on the conclusions or findings. This can be done by describing what changes the sensitivity analyses bring to the interpretation of the data, and whether the sensitivity analyses are more stringent or more relaxed than the primary analysis.

### Some concluding remarks

Sensitivity analyses play an important role is checking the robustness of the conclusions from clinical trials. They are important in interpreting or establishing the credibility of the findings. If the results remain robust under different assumptions, methods or scenarios, this can strengthen their credibility. The results of our brief survey of January 2012 editions of major medical and health economics journals that show that their use is very low. We recommend that some sensitivity analysis should be the default plan in statistical or economic analyses of any clinical trial. Investigators need to identify any key assumptions, variations, or methods that may impact or influence the findings, and plan to conduct some sensitivity analyses as part of their analytic strategy. The final report must include the documentation of the planned or *posthoc* sensitivity analyses, rationale, corresponding results and a discussion of their consequences or repercussions on the overall findings.

## Abbreviations

SA: Sensitivity analysis; US: United States; FDA: Food and Drug Administration; EMEA: European Medicines Association; UK: United Kingdom; NICE: National Institute of Health and Clinical Excellence; RCT: Randomized controlled trial; ITT: Intention-to-treat; PP: Per-protocol; AT: As-treated; LOCF: Last observation carried forward; MI: Multiple imputation; MAR: Missing at random; GEE: Generalized estimating equations; GLMM: Generalized linear mixed models; CHAT: Community hypertension assessment trial; PSA: Prostate specific antigen; CIF: Cumulative incidence function; ESRD: End stage renal disease; IV: Instrumental variable; ANCOVA: Analysis of covariance; SAP: Statistical analysis plan; CONSORT: Consolidated Standards of Reporting Trials.

## Competing interests

The authors declare that they have no competing interests.

## Authors’ contributions

LT conceived the idea and drafted the outline and paper. GW, CHG and MT commented on the idea and draft outline. LM and SZ performed literature search and data abstraction. ZS, LG and CY edited and formatted the manuscript. MM, BD, DK, VBD, RD, VF, MB, JL reviewed and revised draft versions of the manuscript. All authors reviewed several draft versions of the manuscript and approved the final manuscript.

## Pre-publication history

The pre-publication history for this paper can be accessed here:

http://www.biomedcentral.com/1471-2288/13/92/prepub
